# An Atypical Presentation of Bilateral Cavernous Sinus Thrombosis

**DOI:** 10.7759/cureus.64647

**Published:** 2024-07-16

**Authors:** Dominic Spalitto

**Affiliations:** 1 Internal Medicine, University of Kentucky College of Medicine, Bowling Green, USA

**Keywords:** staph aureus, cerebral venous sinus thrombosis (cvst), septic cavernous sinus thrombosis, cerebral vein thrombosis, cavernous sinus thrombosis (cst)

## Abstract

Diagnosis and management of cavernous sinus thrombosis (CST) can be challenging, but several clinical clues can aid in a more time-efficient and cost-effective approach. This condition is rare which can delay diagnosis and be fatal due to the several important neurovascular structures that run through the cavernous sinus. This report discusses a case of CST in a male with substance use disorder whose signs, symptoms, and diagnostic findings were classic for CST. When patients present with multiple concerns, symptom recognition can be challenging, as in this case. Clinicians need to take all symptoms and physical exam findings into consideration and eliminate any bias to provide proper care for patients. Early detection can lead to a more rapid diagnosis and early initiation of adequate treatment to provide better outcomes. There are limited evidence-based guidelines regarding diagnosis and treatment. This report will also review some of the more recent literature on the topic in an attempt to aid healthcare providers in giving proper care to their patients and thereby increasing knowledge and awareness of the subject.

## Introduction

Cavernous sinus thrombosis (CST) is a serious and potentially life-threatening condition. Mortality is highest within 30 days and the main cause of death is transtentorial herniation secondary to large hemorrhagic lesions [1]. This is followed by herniation due to multiple lesions, diffuse brain edema, or diffuse cerebritis [1]. Risk factors for 30-day mortality include depressed consciousness, altered mental status, right hemisphere hemorrhage, and posterior fossa lesions [1]. When it was first reported in 1892, prior to the introduction of antibiotics, it was nearly 100% fatal [2]. Today, mortality has decreased but there is still a risk of long-term sequela. It is characterized by a blood clot in the cavernous sinus which is in the middle cranial fossa near the pituitary gland in the brain. When a clot forms in this area it is considered an emergency, as nearly 30% of cases can be fatal or potentially cause irreversible damage to cranial nerves [3].

The sinus itself contains an array of important cranial nerves and blood vessels including the internal carotid artery and cranial nerves III, IV, V, and VI. The most common symptom is headache; however, the presentation can include orbital signs such as proptosis, reduced visual field acuity, and chemosis as well as other symptoms related to the source of infection like sore throat, otalgia, facial pain, or odontalgia [4]. Focal neurologic deficits are less common, but aphasia and dysarthria have been seen as well. Though the risk of death has decreased due to antibiotics, residual deficits can still occur including permanent blindness, double vision, nerve palsies, and hemiparesis [4]. A careful history and physical exam along with risk factor assessment is important for early diagnosis and treatment to decrease further the risk of death and permanent long-term defects.

## Case presentation

The patient is a 62-year-old male with prior medical history significant for essential hypertension, hyperlipidemia, hypothyroidism, gastroesophageal reflux disease (GERD), and methamphetamine abuse. The patient presented to the emergency department with complaints of chest pain as well as nausea, vomiting, fever, and chills for a week-long duration. He denied headaches as well as presyncope, lightheadedness, syncope, abdominal pain, and dysuria. His chest pain had been intermittent for months; however, it acutely worsened in the last few days.

Vital signs showed a temperature of 101.2 degrees Fahrenheit, a heart rate of 112 beats per minute, a blood pressure of 95/53 mmHg, and a respiratory rate of 18 with 90% oxygen saturation on room air. EKG was unremarkable except for V4, V5, and V6 minimal ST segment depression. Imaging reported pulmonary edema on chest X-ray (CXR) and pleural effusion on chest computed tomographic angiography (CTA). The patient was significantly short of breath but did not require oxygen supplementation. His lab work showed a positive troponin of 0.070ng/ml (range 0.0-0.034ng/ml). He was admitted to the hospital for a congestive heart failure (CHF) exacerbation and suspected non-ST-elevation myocardial infarction (NSTEMI) secondary to hypertension in the setting of substance use. Upon admission, he was started on continuous intravenous (IV) heparin, and he was given IV Lasix®. The IV heparin was monitored using activated partial thromboplastin time (APTT) checks. He did not have a significant response to sublingual nitrate administration, but he admitted to both chest pain and respiratory status improvement with the IV Lasix. His troponins were trended and did not show an increase. Cardiology was consulted and suggested type II NSTEMI as the diagnosis; however, due to risk factors including age, drug abuse, and hypertension a Regadenoson Stress Test and transthoracic echocardiogram were performed. These tests came back with normal findings including a preserved ejection fraction at 55-60%. After the normal stress test, echocardiogram, diuretic treatment, and imaging, the patient’s diagnosis was said to be a type II NSTEMI and CHF exacerbation. Acute coronary syndrome was ruled out, and heparin was discontinued. The patient was having persistent blood pressure in the low 90s systolic and fevers. There was a concern for sepsis due to tachycardia and fever.

A CXR was repeated and showed pulmonary congestion with pulmonary edema which was concerning for inflammation or infection. The patient was negative for COVID-19 and Influenza. Complete blood cell (CBC) count showed mild leukocytosis with neutrophil predominance (Table 1). On hospital day 2, he started to complain of a severe headache. Repeat examination noted that he had drooping of the left eyelid and proptosis, and the patient reported blurry vision. The patient mentioned he did not think these were issues at that time. Stated they had gotten worse. He was started on broad-spectrum antibiotics with IV cefepime and IV vancomycin. For further workup, a head CT, soft tissue neck CT, orbit CT, and head CTA were ordered to assess for intracranial lesions or soft tissue infections of the neck.

**Table 1:** Laboratory analysis during infections workup WBC, white blood cell count; MCV, mean corpuscular volume; ESR, erythrocyte sedimentation rate; BUN, blood urea nitrogen; CRP, C-reactive protein

Parameters	Normal Range (Units)	Patient Results
WBC	4.8-10.8 k/ul	21.8
Hemoglobin	13.0-18.0 g/dl	10.3
MCV	80-94 fl	92
Platelets	140-440 k/ul	187
Segmented Neutrophil %	45.0-70.0%	87
Band Neutrophil %	1-5%	10
Lymphocytes %	25.0-40.0%	2
Eosinophil %	0-3%	1.8
Basophil %	0-1.0%	0.3
Neutrophil number	1.90-8.00 k/ul	18.91
Immature granulocyte number	0.00-0.05 k/ul	0.45
Lymphocyte number	0.90-5.00 k/ul	0.66
Monocyte number	0.16-1.0 k/ul	1.60
Eosinophil number	0.0-0.80 k/ul	0.01
ESR	0-20 mm/hr	123
Sodium	137-145 mmol/l	138
Potassium	3.5-5.1 mmol/l	4.1
Chloride	98-107 mmol/l	100
Sodium bicarbonate	22-30 mmol/l	26
BUN	9-20 mg/dl	18
Creatinine	0.66-1.42 mg/dl	0.90
Lactic acid	0.7-2.0 mmol/l	3
CRP	0-1.0 mg/dl	20.9
Procalcitonin	ng/ml	7.830

**Figure 1:**
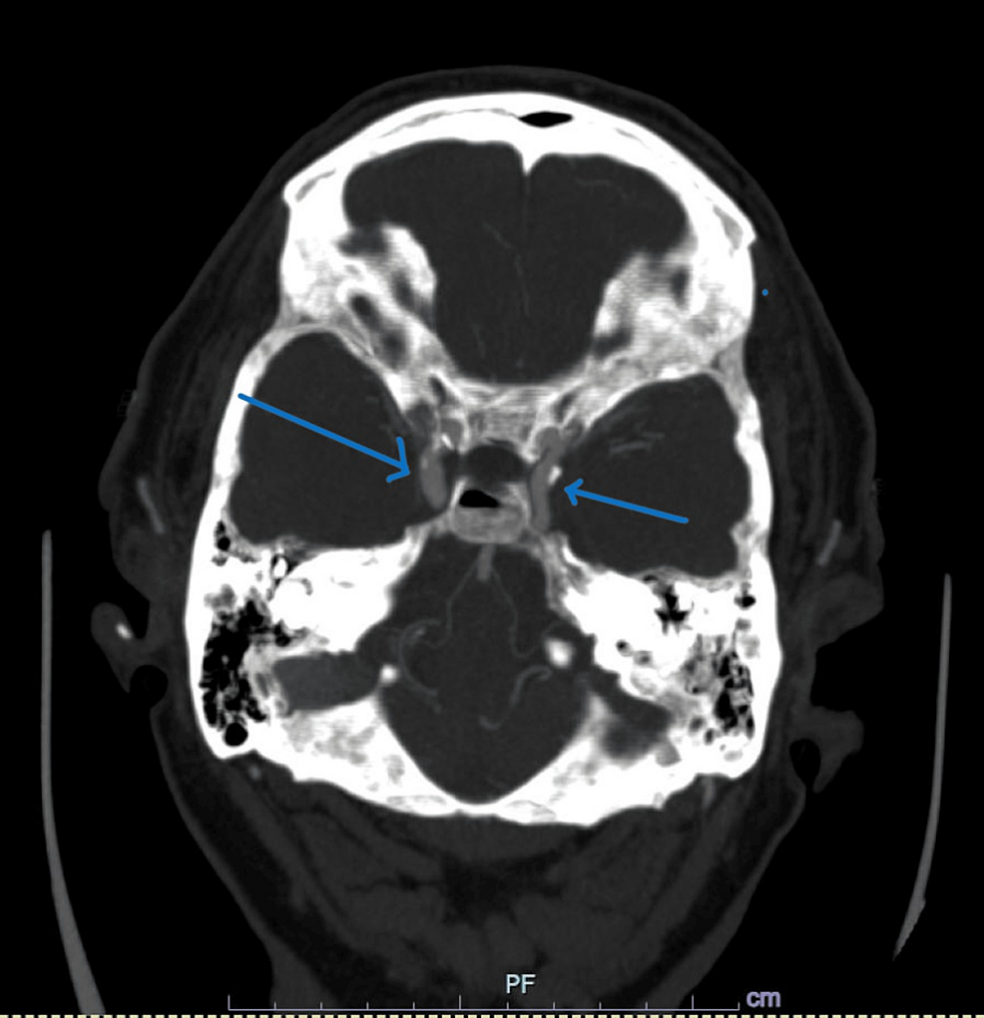
CTA of the head showing enlargement of the ophthalmic veins CTA, computerized tomographic angiography

**Figure 2:**
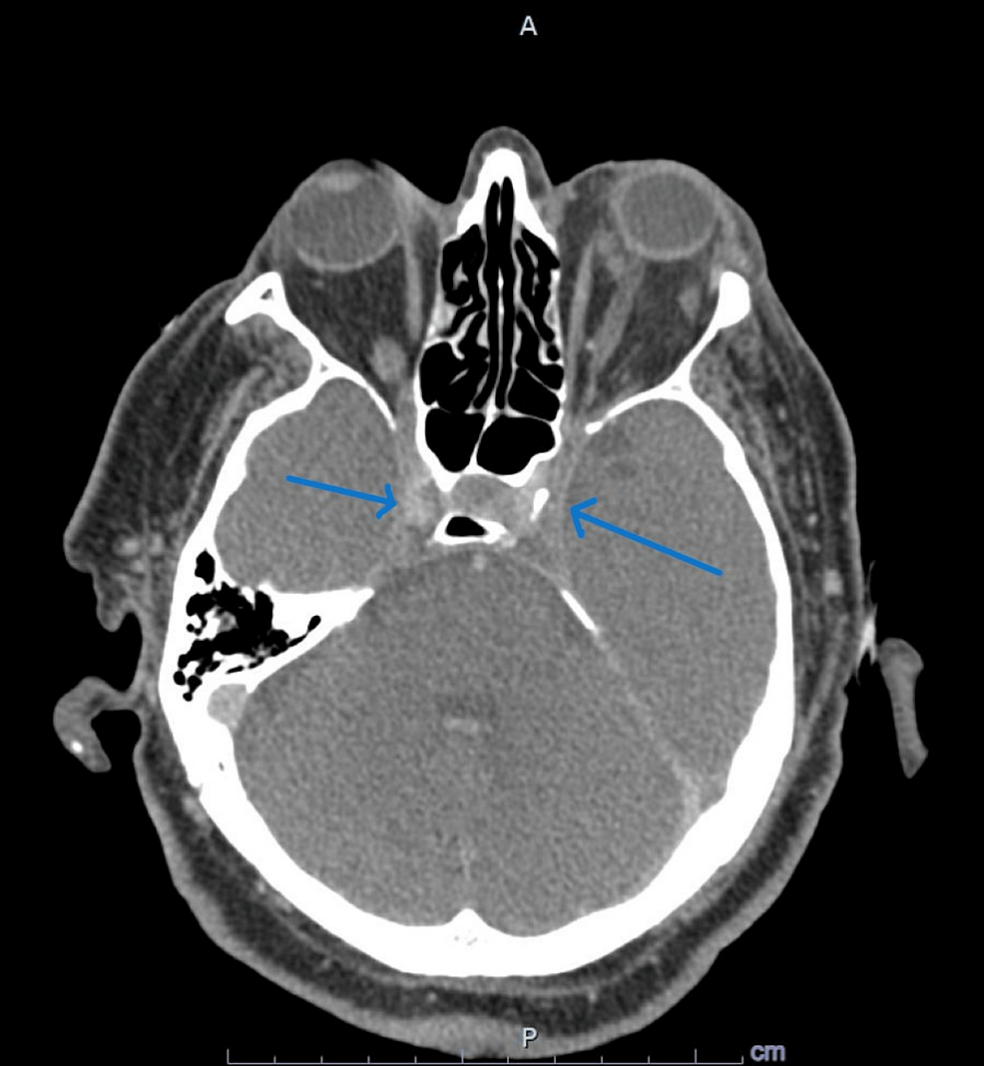
CT venogram of the head showing dilated ophthalmic veins with diminished enhancement in both cavernous sinuses compatible with bilateral cavernous sinus thrombosis

His infectious workup resulted in elevated lactic acid, CRP, ESR, WBC, and procalcitonin (Table 1). His pneumonia polymerase chain reaction (PCR) was positive for *Streptococcus *species and his blood cultures revealed *Streptococcus anginosus*. The original head CT was unremarkable, and the CTA head showed enlarged ophthalmic veins (Figure 1). Further investigation with a CT venography of the head confirmed the diagnosis of bilateral CST (Figure 2). The radiologic read reported bilateral exophthalmos left greater than right, thrombus formation in each ophthalmic vein, bilateral CST, and thrombus formation in the right internal jugular vein. IV heparin was restarted and monitored with APTT. Ophthalmology was consulted and agreed on anticoagulation and antibiotic treatment. He was found to have increased intraocular pressure and was started on Cosopt®, brimonidine, and latanoprost eye drops. The patient was having persistent and worsening headaches and had not yet shown clinical improvement. The Ophthalmology team recommended transfer to a facility that had oculoplastic services in case orbital decompression becomes necessary. The patient had been adequately treated from a cardiac standpoint and his CHF exacerbation had resolved. He was on anticoagulation, broad-spectrum antibiotics, and in stable condition. Per the Ophthalmologist's recommendation, he was transferred to a higher level of care facility.

## Discussion

CST makes up about 1-4% of all cerebral venous and sinus thrombosis [5]. Due to the scarcity of cases, it has been hard to estimate incidence. In general, cerebral venous thrombosis only occurs at about one per 100,000 annually [5]. The cavernous sinus is the least common location for cerebral venous thrombosis [5]. Historically, it was thought that CST/cerebral venous thrombosis occurs more commonly in children. Recent studies have challenged this belief. A cross-sectional study conducted in 2012 looked at prior data and new literature. They concluded that the rates of cerebral venous thrombosis can be comparable to those of bacterial meningitis, and CST can be nearly twice as common in adults than in children [6]. This is important because the incidence of CST/cerebral venous thrombosis is probably higher amongst adults than previously believed. Even with the advent of antibiotics, morbidity remains high, and complete recovery is seldom with nearly one sixth of individuals affected left with some degree of visual impairment and nearly half of the individuals left with cranial nerve defects [7].

CST typically presents with severe headache, tearing, swelling/irritation around one or both eyes, drooping eyelids, inability to move eyes, high fever, fatigue, vision loss, seizures, or altered mental status [8]. Ocular symptoms commonly occur first due to the presence of cranial nerves III, IV, V, and VI that run through the cavernous sinus with the sixth cranial nerve most frequently affected [4]. The sixth cranial nerve is closer to the internal carotid artery and they run more medially while the other cranial nerves are more lateral and protected by a thick layer of dura [4]. These cranial nerves aid in extra ocular movements, and the fifth cranial nerve also functions in sensations of the face. Compression of these structures can result in periorbital edema, proptosis, diplopia, loss of vision, ophthalmoplegia, and paresthesias around the orbit and face [8]. Blindness can result in 8% to 15% of patients [8].

Contrast-enhanced CT or an MRI of the head is optimal, however, not as ideal as a CT venogram and contrast-enhanced MR venogram [8]. A non-contrast CT may show subtle abnormalities like dilation of the ophthalmic veins or exophthalmos and can rule out bleeding or masses, however, can miss a CST. A contrast-enhanced MRI would show the bulging of the cavernous sinus, increased dural enhancement, and absent flow [8]. The most sensitive test of choice would be the CT or MR venogram [8]. Venography allows for visualization of significant findings such as carotid artery narrowing, carotid wall enhancement, cerebral infarcts, meningitis, or hemorrhages.

The most common causes of CST are typically due to non-infectious causes like trauma or surgery or from infectious pathology. Infectious causes are more common. Sinusitis, otitis, odontogenic sources, facial or orbital cellulitis/abscesses, or mastoiditis are examples of infectious causes [1,4,8]. One study suggested the most common cause was sinusitis of the sphenoid sinus [4]. Most of the infectious causes stem from the "Danger Triangle of the face", which is from the corners of the mouth to the bridge of the nose. *Staphylococcus aureus* accounts for nearly 67% of the cases [8] and is said to be the most common cause [9].

Due to the scarcity of the disease, there has been no guideline-directed treatment algorithm. There are also no random controlled trials to guide treatment. Literature frequently discusses antibiotics, steroids, and anticoagulation, but there is limited research on interventional therapies. Studies show that antibiotics and anticoagulation are beneficial; however, steroids are of equivocal benefit [9]. Steroids might decrease inflammation and vasogenic edema, however, has not demonstrated efficacy [8]. Most experts currently recommend antibiotic therapy with an agent that covers methicillin-resistant *Staphylococcus*, a third-generation cephalosporin, and metronidazole with consideration for antifungal treatment in immunocompromised patients [8,9]. Immunocompromised conditions include patients with connective tissue disorders, malignancies, immunosuppressants, or patients with transplanted organs [9]. Antibiotics are de-escalated with the isolation of causative organisms and are given for about three to four weeks or at least two weeks beyond clinical resolution [8]. There are no current recommended surgical interventions; however, source control is needed. If there is an abscess or infected bone then they should be removed.

Anticoagulation is given in the absence of strong contraindications and extends several weeks to months [8,9]. It has been documented retrospectively that mortality decreases from 40% to 14% with the use of unfractionated weight heparin (UFWH), and a reduction in morbidity from 61% to 31% when anticoagulation is combined with antibiotics [8]. The European Federation of Neurological Societies mentions that three months of anticoagulation in cerebral venous and sinus thrombosis should be efficient to decrease the risk of further injury. There are risks and benefits to anticoagulation. The benefits would be to stop the progression of thrombosis and prevent clot propagation, but the risk would be intracranial bleeding [9]. Currently, it is suggested that the reduced mortality and morbidity benefit outweighs the risk.

The case discussed above details a patient who presented with chest pain and shortness of breath. He was found to have a diastolic heart failure exacerbation. Vital signs and lab analysis hinted towards infection. On initial assessment, certain physical exam findings and symptoms were not accounted for. He was found to have symptoms that suggested infection from a possible intracranial pathology. Symptoms included proptosis, blurry vision, and headache while objective findings showed leukocytosis, tachycardia, and fever. Blood cultures were obtained which were positive for *S. anginosus*, and imaging confirmed the diagnosis of bilateral CST. This case is unique in that the patient did not have any of the presenting common causes like sinusitis, or odontogenic infections, nor did he have a recent history of head trauma or facial surgery. It was said that the patient was high risk secondary to his use of drugs and this contributed to his bacteremia and eventual CST. He was treated appropriately with antibiotics and anticoagulation. Due to the acuity of his condition, he was transferred to a facility that had oculoplastic services for further management.

## Conclusions

Clinicians should have a high index of suspicion for CST when patients present with headaches, pain with ocular movements, visual changes, proptosis, chemosis, and cranial nerve III, IV, V, and VI changes. Fever with a recent history of head infections or trauma are also concerning findings. Antibiotics decrease the risk of mortality, and with the advancement in technology paired with increased awareness, the diagnosis of CST has become more frequent. Further investigation is warranted to discover finer treatment algorithms or modalities to provide better quality care and decrease the mortality rate and long-term sequelae. This case was remarkable in that the patient did not have any of the classic causes like orbital trauma, infections in the danger triangle, or recent surgical intervention. This stresses the importance of increased awareness, diligent physical exams by assessing all aspects including cranial nerve functions, and recognition of early symptoms. This can lead to rapid diagnosis and early treatment.
